# ATTED-II in 2018: A Plant Coexpression Database Based on Investigation of the Statistical Property of the Mutual Rank Index

**DOI:** 10.1093/pcp/pcx191

**Published:** 2017-12-04

**Authors:** Takeshi Obayashi, Yuichi Aoki, Shu Tadaka, Yuki Kagaya, Kengo Kinoshita

**Affiliations:** 1 Graduate School of Information Sciences, Tohoku University, 6-3-09, Aramaki-Aza-Aoba, Aoba-ku, Sendai, 980-8679 Japan; 2 Tohoku Medical Megabank Organization, Tohoku University, Sendai, 980-8573 Japan; 3 Graduate School of Medicine, Tohoku University, Sendai, 980-8573 Japan; 4 Institute of Development, Aging, and Cancer, Tohoku University, Sendai, 980-8575 Japan

**Keywords:** Arabidopsis, Comparative transcriptomics, Database, Gene coexpression, Gene network, Statistics

## Abstract

ATTED-II (http://atted.jp) is a coexpression database for plant species to aid in the discovery of relationships of unknown genes within a species. As an advanced coexpression analysis method, multispecies comparisons have the potential to detect alterations in gene relationships within an evolutionary context. However, determining the validity of comparative coexpression studies is difficult without quantitative assessments of the quality of coexpression data. ATTED-II (version 9) provides 16 coexpression platforms for nine plant species, including seven species supported by both microarray- and RNA sequencing (RNAseq)-based coexpression data. Two independent sources of coexpression data enable the assessment of the reproducibility of coexpression. The latest coexpression data for Arabidopsis (Ath-m.c7-1 and Ath-r.c3-0) showed the highest reproducibility (Jaccard coefficient = 0.13) among previous coexpression data in ATTED-II. We also investigated the statistical basis of the mutual rank (MR) index as a coexpression measure by bootstrap sampling of experimental units. We found that the error distribution of the logit-transformed MR index showed normality with equal variances for each coexpression platform. Because the MR error was strongly correlated with the number of samples for the coexpression data, typical confidence intervals for the MR index can be estimated for any coexpression platform. These new, high-quality coexpression data can be analyzed with any tool in ATTED-II and combined with external resources to obtain insight into plant biology.

## Introduction

Gene coexpression analysis, which is a guilt-by-association approach based on gene expression profiles, can uncover functionally related gene pairs. Of particular importance, coexpression information based on a large amount of publicly available transcriptome data is not highly affected by a specific experimental condition and provides a fundamental view of gene functional networks. A number of databases have been developed to provide condition-independent coexpression information for various applications, from gene prioritization to the delineation of global relationships among multiple network modules (Aoki et al. 2007, Usadel et al. 2009, Rung and Brazma 2013). In addition, comparisons of gene networks among multiple species can be used to identify conserved and specific gene modules in an evolutionary context. Conserved gene relationships suggest a core function for a cell system, whereas species-specific relationships are linked to the differentiation of species (Stuart et al. 2003, Oti et al. 2008, Obayashi and Kinoshita 2011, Movahedi et al. 2011, Okamura et al. 2015, Ruprecht et al. 2017).

Although the importance of coexpression data is widely recognized, related methodologies have various limitations. For example, the determination of coexpression consists of multiple steps, from the preparation of gene expression data to the calculation of a gene-to-gene matrix (Zhang and Horvath 2005). To optimize this procedure, we should find the optimal combination of algorithms for each step. In the case of the detection of differentially expressed genes from microarray or RNA sequencing (RNAseq) data, which is related to the task of coexpression calculation, we can use a benchmark dataset, such as MicroArray Quality Control datasets (Canales et al. 2006, Kadota et al. 2009). However, determining a gold standard for gene coexpression is quite difficult because gene coexpression is a summarization metric of gene expression data and thus it depends on the composition of individual experimental samples (Usadel et al. 2009).

We have been continuously developing a coexpression database for plants, ATTED-II (http://atted.jp). In previous reports, we have described associations between gene coexpression and *cis*-elements (Obayashi et al. 2007), the development of a coexpression measure with sample weights and the mutual rank (MR) index (Obayashi et al. 2009), the analysis of condition-specific coexpression (Obayashi et al. 2011) and the evaluation of coexpression (Obayashi et al. 2014). In the most recent report associated with ATTED-II, we demonstrated the detection of lineage-specific gene coexpression using eight coexpression platforms for four species (Aoki et al. 2016b). However, we could not apply statistical tests to the identification of lineage-specific coexpression for two reasons. First, the correspondence of sample conditions among the four species was not clear. To account for potential differences in the compositions of samples, we focused on lineage-specific coexpression, rather than species-specific coexpression, in which detected coexpression was also supported by conserved coexpression relationships. Secondly, statistical characteristics of the coexpression index were not clear. We have adopted the MR index of gene-to-gene correlations as a coexpression measure because it has a higher predictive power for gene function than the Pearson’s correlation coefficient (PCC) (Obayashi and Kinoshita 2009). However, the MR index was not statistically characterized, thereby restricting the usage of this index for meta-analyses of coexpression data. Here, we report an update of ATTED-II that focuses on the following three aims to promote meta-coexpression analyses among species: (i) establishment of comparable assessment measures of gene coexpression for different species; (ii) investigation of the statistical properties of the MR index; and (iii) construction of high-quality coexpression data. As a result of these revisions, ATTED-II provides multispecies coexpression data with improved accuracy and usability.

## Results and Discussion

### New ATTED-II coexpression data for nine species

We updated both the microarray-based and RNAseq-based coexpression data in ATTED-II (**Table 1**), based on Affymetrix GeneChip data in ArrayExpress (Kolesnikov et al. 2015) and Illumina RNAseq data in the DNA Data Bank of Japan (DDBJ) (Ogasawara et al. 2013). We also added two sets of RNAseq-based coexpression data for barrel medick (*Medicago truncatula* RNAseq; Mtr-r) and grape (*Vitis vinifera* RNAseq; Vvi-r). As a result, ATTED-II provides two coexpression platforms for each of nine species, except for field mustard (*Brassica rapa*) and poplar (*Populus trichocarpa*) for which only a single platform is available. Multiple platforms for individual species are preferable for determining the reproducibility of coexpression data, as shown below. These coexpression data are available in the search or draw tools in ATTED-II. To assist users, examples of queries for every species are provided (http://atted.jp/top_search.shtml and http://atted.jp/top_draw.shtml).

**Table 1:** Coexpression data in ATTED-II version 9

Species	Platform ID^a^	Version	Genes	Samples	Logit-MR error	Function score^b^	Reproducibility^c^
*Arabidopsis thaliana*	Ath-m	c7.1	20,819	16,033	0.37	5.43	0.136
	Ath-r	c3.0	22,760	2,120	0.71	5.17	
*Brassica rapa*	Bra-r	c2.1	28,978	188	1.04	4.77	–
*Glycine max*	Gma-m	c3.1	15,746	1,131	0.74	3.37	0.076
	Gma-r	c3.0	8,373	599	1.02	7.64	
*Medicago truncatula*	Mtr-m	c3.1	20,376	975	1.04	4.43	0.021
	Mtr-r	c1.1	3,753	41	1.46	2.65	
*Oryza sativa*	Osa-m	c6.1	19,867	2,250	0.76	4.98	0.041
	Osa-r	c2.1	24,437	336	1.07	4.06	
*Populus trichocarpa*	Ppo-m	c2.1	21,910	765	1.10	3.82	–
*Solanum lycopersicum*	Sly-m	c2.1	5,721	401	1.04	4.08	0.041
	Sly-r	c2.1	20,564	282	1.01	3.87	
*Vitis vinifera*	Vvi-m	c3.1	9,421	314	1.14	4.47	0.028
	Vvi-r	c1.1	18,587	346	0.90	3.10	
*Zea mays*	Zma-m	c3.1	10,777	806	1.11	4.62	0.055
	Zma-r	c2.1	32,274	1,794	0.88	4.42	

^a^Xxx-m, microarray-based coexpression; Xxx-r, RNAseq-based coexpression.

^b^Predictive performance of the KEGG annotation represented by partial AUROC (1E-04). A higher score indicates a better performance.

^c^Jaccard coefficient for common edges between the platforms in the same species. The top three coexpressed genes from every gene were used as edges.

### Reproducibility of co expression data

Determining the reproducibility of coexpression data is straightforward. First, we checked microarray-based and RNAseq-based coexpression data for Arabidopsis, with the longest history of coexpression data in ATTED-II. **Table 2** shows overlap in the coexpression edges between each data type, i.e. microarray-based Arabidopsis coexpression data (Ath-m) and RNAseq-based data (Ath-r). This table clearly shows the successful development of Arabidopsis coexpression data in ATTED-II, with the highest Jaccard coefficient between the current coexpression data, Ath-m.c7-1 and Ath-r.c3-0. Reproducibility results for the other species are summarized in **Table 1**. In addition to the highest level of reproducibility with the Arabidopsis data, data for soybean (*Glycine max*) and maize (*Zea mays*) were relatively highly reproducible, whereas coexpression data for *M. truncatula* and *V. vinifera* were not consistent among data types within each species, suggesting that one or both of the coexpression datasets (i.e. microarray and RNAseq) is of low quality.

**Table 2:** Jaccard coefficients of common edges among a series of coexpression data for Arabidopsis in ATTED-II

	Ath-r.c3-0	Ath-r.c2-0	Ath-r.c1-0
Ath-m.c7-0	0.134	0.055	0.038
Ath-m.c6-0	0.111	0.057	0.040
Ath-m.c5-0	0.106	0.056	0.040
Ath-m.c4-1	0.078	0.046	0.032
Ath-m.c3-1	0.061	0.042	0.029

Xxx-m, microarray-based coexpression; Xxx-r, RNAseq-based coexpression.

Note that a Jaccard coefficient of 1 indicates complete overlap between the two sets of coexpression edges, whereas a Jaccard coefficient of 0 indicates no overlap.

### Consistency of gene co expression data with gene function

**Fig. 1:**
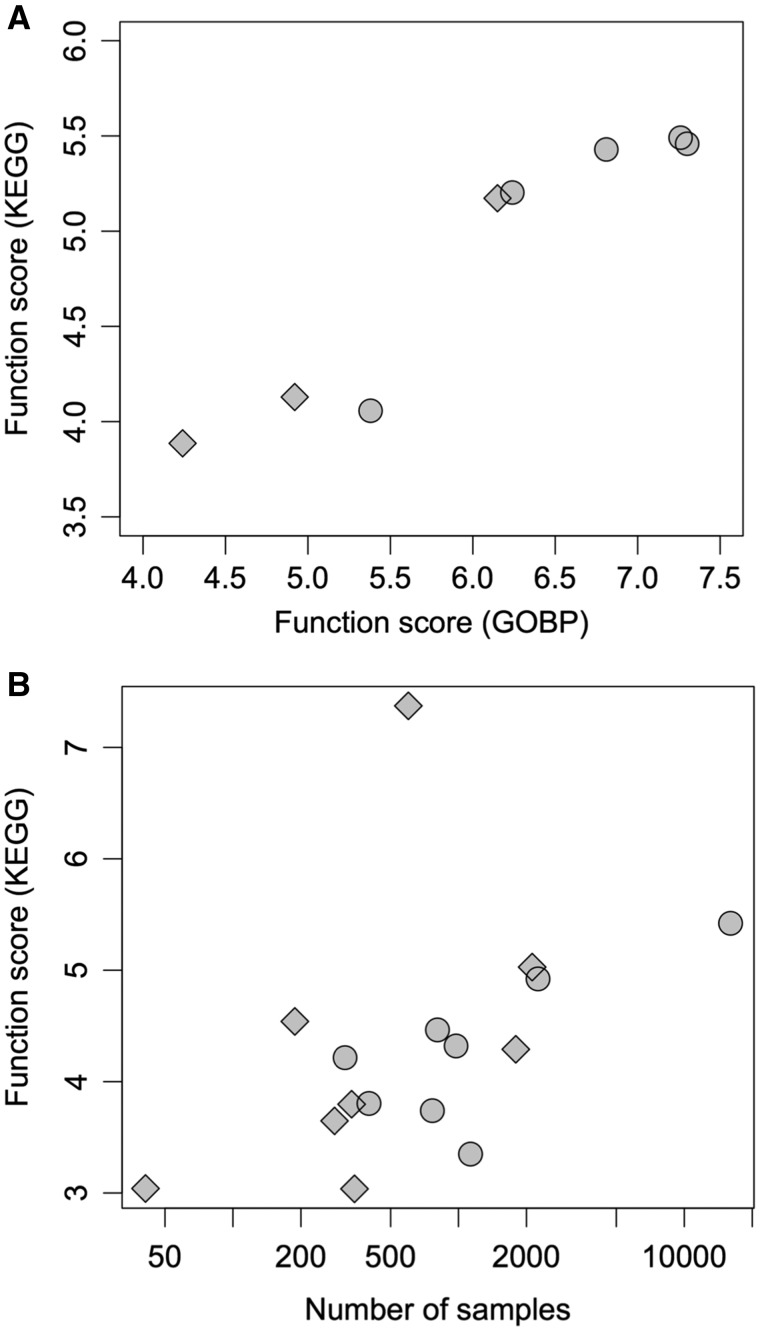
Quality assessment based on the consistency of known gene functions. As a measure of the quality of gene coexpression data, the power to discriminate gene pairs sharing a common functional annotation from other gene pairs was used. (A) Previous and current Arabidopsis coexpression data were assessed by this discrimination analysis. Irrespective of the gene annotation source (GO Biological Process or KEGG pathway), the quality trend was consistent. (B) The current 16 coexpression platforms were assessed with the discrimination analysis using the KEGG pathway annotation. Circles and diamonds indicate microarray-based and RNAseq-based coexpression datasets, respectively.

Although reproducibility can be measured without any external data, this measure mutually depends on the coexpression datasets. In other words, this index does not indicate the quality of individual gene coexpression data. Another concern is that it is possible to select a limited set of samples to achieve highly reproducible coexpression data. In such cases, high reproducibility does not indicate high quality. As another quality measure, the enrichment of functional annotations in coexpressed genes is useful because gene coexpression information is used to identify functionally related genes. Gene Ontology (GO) annotation provides a rich resource for the functional annotation of genes (Gene Ontology Consortium 2015). Conveniently, the directed acyclic graph structure of GO terms enables the selection of terms that have a particular range of information content (Lord et al. 2003). However, GO annotations are not sufficient for plant species in ATTED-II, except Arabidopsis, and thus cannot be used for the quality assessment of coexpression data among species (Obayashi et al. 2014). To resolve this problem, we adopted the KEGG (Kyoto Encyclopedia of Genes and Genomes) pathway annotation (Kanehisa et al. 2016) as an alternative method. To select highly informative annotations, we excluded pathways associated with >100 genes in a genome, resulting in 102.2 pathways, on average, for the nine species. To characterize the selected KEGG pathway annotations, we first checked the consistency of the KEGG evaluation results with the GO evaluation results using a series of coexpression data for Arabidopsis. The discriminative power based on gene pairs with and without common gene function annotations showed a similar trend between the two annotation types (GO and KEGG), supporting the validity of KEGG pathway annotation for the quality assessment of coexpression (Fig. 1A). Because KEGG pathway annotations are provided for a broad range of species with almost the same annotation density across species, this will be useful not only for comparisons among plant species, but also for comparisons of coexpression data across species in distinct kingdoms, such as microalgae and animals (Okamura et al. 2015, Aoki et al. 2016a).

Using the function score based on the KEGG pathway annotation, the efficiency of coexpression for all coexpression platforms was compared (**Table 1**; Fig. 1B). The function scores were correlated with the logarithmic number of samples for each species (PCC = 0.44), as reported previously (Ballouz et al. 2015). These trends were observed for both microarray- and RNAseq-based data. The only exception was Gma-r, which showed a high score, despite a relatively small number of samples. In this functional scoring, we did not eliminate paralogous gene pairs, which usually have similar functional annotations and expression profiles, resulting in overestimation of scores in species having a large number of paralogous genes. Furthermore, the function score is based on unevenly distributed annotations across genes in a species. For example, highly expressed genes tend to be well studied and thus well annotated (**Supplementary Fig. S1**). Therefore, the function score for coexpression may not reflect the overall performance of coexpression data. Nonetheless, the KEGG scores for each species were generally consistent with the reproducibility scores (**Table 1**). As described above, reproducibility is mutually dependent, and thus a lower quality of coexpression data for a species mainly limits the reproducibility score. In fact, reproducibility scores were well correlated with the smaller KEGG scores across the two platforms in a species (PCC = 0.82).

### MR as a statistical indicator

**Fig. 2:**
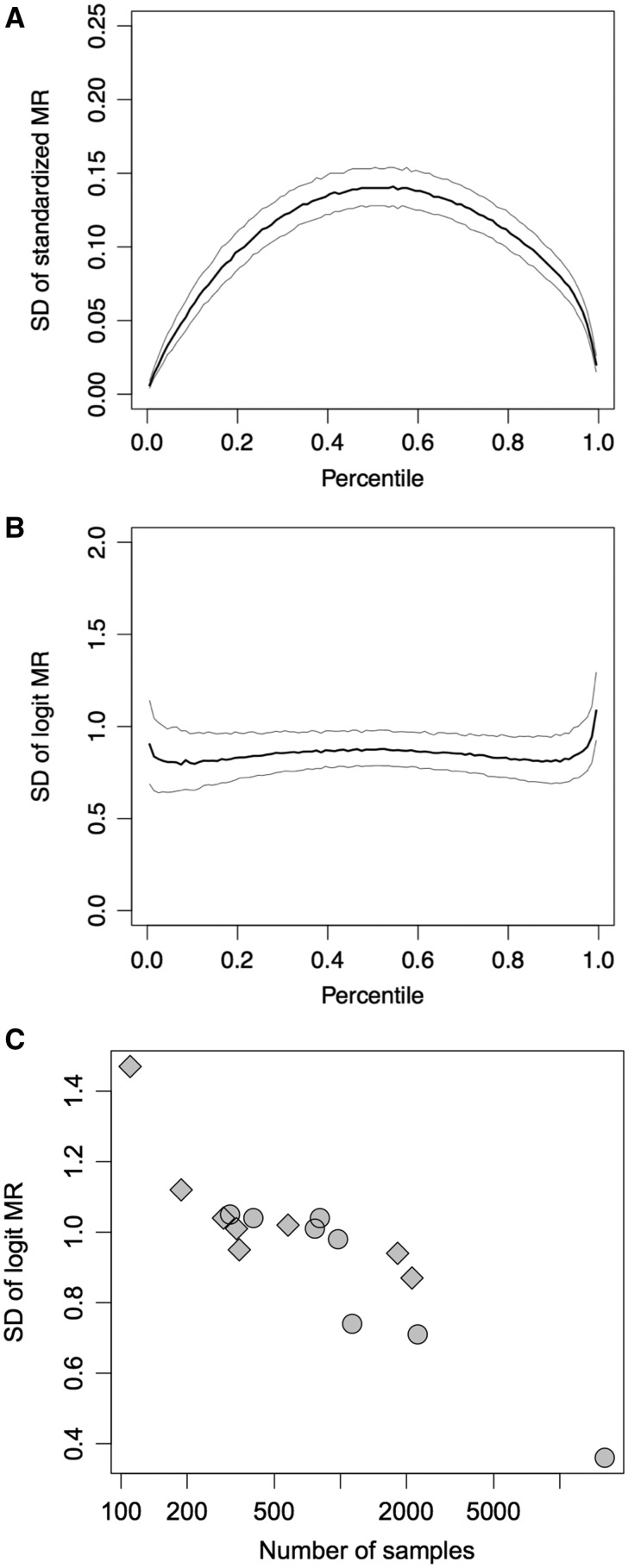
Properties of the error distribution of MR and logit-MR values. SDs of the bootstrapped MRs from RNAseq-based Arabidopsis coexpression data (Ath-r.c3-0) are shown. (A, B) SDs of bootstrapped MR values (A) and of logit-MR values (B) are shown against the mean of the bootstrapped MR values. The black lines show the median values with a sliding window corresponding to the 0.01 percentile of the MR without overlap, whereas the gray lines represent the first and third quantiles. (C) Mean SDs for the current 16 coexpression platforms in ATTED-II are plotted against the number of samples for each platform. Circles and diamonds indicate microarray-based and RNAseq-based coexpression datasets, respectively.

We previously reported that the MR index is a powerful indicator of the co-function of a gene pair (Obayashi and Kinoshita 2009). However, the poor characterization of this index limits downstream analyses. To investigate the distribution and confidence interval of the MR index, we repeatedly calculated the coexpression matrix from bootstrapped samples of the experimental unit. Fig. 2A shows the SD of the MR index for 100 sets of bootstrapped RNAseq expression data in Arabidopsis. The horizontal axis represents the mean of 100 bootstrapped MR values expressed as a percentile from a positive correlation (small MR) to a negative correlation (large MR). The MR ranges from ‘1’ to ‘the number of genes minus 1’ (e.g. 1–22,759 for the Ath-r.c3-0 platform, which includes 22,760 genes). The vertical axis represents the median (black curved line) and the first and third quantiles (gray curved lines) of the SD of the bootstrapped MR values. The MR index was precise near each end of its range, but in the middle of the range, indicating no correlation, MR was easily affected by chance (Fig. 2A). Additionally, the skewness and kurtosis of the error distribution indicated that the data were not normally distributed (**Supplementary Fig. S2A**, **B**). In fact, this non-normal distribution is commonly observed in the PCC (*r*) of samples, which can be converted to a normal distribution using a logit transformation known as the Fisher transformation (Fisher 1915). This logit transformation can also be applied to the range-standardized MR index (0 < *p*_*i*_ < 1), which is calculated by dividing by the total number of genes (*N*), $p_{i}=MR_{i}/N$.

(1) $$\text{log}\text{i}\text{t}(p_{i})=\text{log}(\frac{p_{i}}{1-p_{i}})$$

Note that this transformation is equivalent to the Fisher transformation with the range standardization of *r* as $p_{i}=(r+1)/2$. Fig. 2B shows the distribution of SD for the logit-transformed MR values (hereafter, logit-MR error). The logit-MR error was nearly constant across the entire range of MR values. Along with the skewness and kurtosis of the distribution (**Supplementary Fig. S2C**, **D**), the logit-MR error can be regarded as a normal distribution with a constant SD for the entire range of MR values. In the case of Ath-r.c3-0, shown in Fig. 2B, the average logit-MR error was 0.87. The logit-MR errors varied across the coexpression platforms (**Table 1**) and were strongly correlated with the logarithmic number of samples for each coexpression platform (PCC = −0.90) (Fig. 2C). This characteristic is useful for estimating confidence intervals for the MR index solely based on the number of samples. For example, the logit-MR error for coexpression data based on 500 samples will result in a value of approximately 1. **Table 3** shows the 90% confidence intervals for typical MR values with logit-MR error = 1 or 0.5. ATTED-II shows the top 300 coexpressed genes as the default. Taking into account the confidence intervals of the MR index, this list typically includes most of the top 100 coexpressed genes. Note that the logit-MR error on the rightmost edge of the MR percentile is slightly larger than that on the leftmost edge (Fig. 2B), suggesting that negative coexpression relationships are more unstable than positive relationships in the MR calculation.

**Table 3:** The 90% confidence intervals of typical MR values with different error levels

MR	Bootstrap SD = 0.5		Bootstrap SD = 1	
1	0.8	1.4	0.7	2.1
3	1.9	4.9	1.3	8.3
10	5.9	17.3	3.5	30.2
30	14.2	52.6	6.6	92.5
100	56.9	175.8	32.4	308.4

### Integration of multiple MR values

Based on the normality of the logit-transformed MR values, we can integrate multiple MR values. In general, the arithmetic average can be generalized using the transformation function f and its reverse function $f^{-1}$.

(2) $$Average_{F}=f^{-1}(\sum f(x_{i})/\sum i)$$

For example, using identity, logarithmic and rational functions as the transformation functions results in arithmetic, geometric and harmonic averages, respectively. Here, to determine the average with a logit transformation, we adopted a logit function for f and a sigmoid function for $f^{-1}$. For *k* MR values (*MR*_1_, *MR*_2_, …, *MR*_*k*_) with individual weights (*w*_1_, *w*_2_, … *w*_*k*_; Σ*w*_*i*_ = 1), the weighted logit average of the MR values is as follows:

(3) $$MR_{average}=\frac{N\Pi MR_{i}^{w_{i}}}{\Pi (N-MR_{i}{)}^{w_{i}}+\Pi MR_{i}^{w_{i}}}$$

Note that this equation can be approximated as a weighted geometric mean under *MR*_*i*_ << *N*.

(4) $$MR_{geometricmean}≃\Pi MR_{i}^{w_{i}}$$

In ATTED-II, the CoExSearch tool provides an integrated list of coexpressed genes for multiple gene queries (http://atted.jp/top_search.shtml#CoExSearch). This tool uses equal weights ($w_{i}=1/k$) from Eqn. 3, simplified as follows:

(5) $$MR_{average}=\frac{N\sqrt[{k}]{\Pi MR_{i}}}{\sqrt[{k}]{\Pi (N-MR_{i})}+\sqrt[{k}]{\Pi MR_{i}}}$$

Even given the high manageability of logit-transformed MR values, we have retained the original MR index in the ATTED-II database. Because the MR index is derived from the ranking of the coexpression strengths, this index implies the maximum size of the coexpression network including the guide gene and its coexpressed genes.

### Slight, but stable improvement in coexpression quality based on the bootstrapping procedure

Sample bootstrapping is widely used to improve the generalization ability of a model (Breiman 1994). As we proposed to use the average method of the MR index, we could use the average of the bootstrapped MR values as a coexpression index. As a result, the bootstrapping and average approach (a simple bagging approach) returned substantially better results than the original coexpression dataset using all samples at once (‘Bagging effect’ in **Table 4**). Although the gains from the bagging procedure were not large for most coexpression platforms, this procedure resulted in relatively greater gains for platforms with a smaller number of samples. The PCC between the bagging effect and the logarithmic number of samples was −0.42, indicating that the bagging procedure was not effective for platforms that include larger numbers of samples. This may be explained by the limited number of bootstrap replicates in our procedure (i.e. 100).

**Table 4:** Comparison of calculation methods

Platform	No. of genes	No. of samples	Q	CQ	CQB	Combat effect	Bagging effect
Ath-m	20,819	16,033	5.46	5.42	5.43	0.99	1.00
Gma-m	15,746	1,131	3.29	3.35	3.37	1.02	1.01
Mtr-m	20,376	975	3.41	4.32	4.43	1.27	1.03
Osa-m	19,867	2,250	4.60	4.92	4.98	1.07	1.01
Ppo-m	21,910	765	3.49	3.74	3.82	1.07	1.02
Sly-m	5,721	401	3.31	3.80	4.08	1.15	1.07
Vvi-m	9,421	314	3.93	4.22	4.47	1.07	1.06
Zma-m	10,777	806	4.00	4.46	4.62	1.12	1.03
Ath-r	22,760	2,120	4.56	5.12	5.17	1.12	1.01
Bra-r	28,978	188	4.54	4.63	4.77	1.02	1.03
Gma-r	8,373	599	6.56	7.60	7.64	1.16	1.01
Mtr-r	3,753	41	2.54	2.62	2.65	1.03	1.01
Osa-r	24,437	336	3.43	3.80	4.06	1.11	1.07
Sly-r	20,564	282	3.55	3.70	3.87	1.04	1.05
Vvi-r	18,587	346	3.21	3.07	3.10	0.96	1.01
Zma-r	32,274	1,794	3.98	4.35	4.42	1.09	1.01

Q, quantile normalization; CQ, ComBat-Quantile normalization; CQB, Bagging procedure for ComBat-Quantile normalized expression data.

### Batch normalization of gene expression data

Batch effects are a major source of technical noise in transcriptome data (Leek et al. 2010, Goh et al. 2017). In the previous coexpression calculation procedure in ATTED-II, we applied batch zero-centering (Usadel et al. 2009), which standardizes the mean expression level of each gene in each experiment to zero (Sims et al. 2008). On the other hand, in our previous investigation, direct standardization of variances of each gene in each experiment did not improve the quality of coexpression results, probably due to loss of fold-change information in gene expression. ComBat is a method that can be used to estimate stably the mean and variance of the batch effect by the empirical Bayes method (Johnson et al. 2007) and is effective for transcriptome and coexpression analyses (Müller et al. 2016, Vandenbon et al. 2016). However, batch estimation for data with an unbalanced batch-group design risks introducing another bias (Nygaard et al. 2016). Because the expression data for coexpression calculation are aggregated from different experiments, this is a case of the unbalanced batch-group design. Therefore, we investigated the effect of variance standardization by the empirical Bayes estimation in addition to batch zero-centering for a set of the current coexpression platforms in ATTED-II (**Table 4**). As a result, batch normalization with gene- and experiment-specific noise variances had notable effects on microarray-based and RNAseq-based coexpression data (‘ComBat effect’ in **Table 4**). Based on this finding, we incorporated the ComBat normalization method into the latest calculation pipeline in ATTED-II.

## Materials and Methods

### Construction of gene coexpression data

In ATTED-II version 9, the coexpression calculation procedure was slightly modified from the previous procedure to reduce the calculation cost sufficiently to enable bootstrapping trials for multiple species. For microarray-based coexpression data, we downloaded the GeneChip CEL files from ArrayExpress (Kolesnikov et al. 2015), normalized them based on the RMA method (Irizarry et al. 2003) and applied ComBat normalization (Johnson et al. 2007) using an experimental unit as a batch. Before calculating correlations among all probe pairs, we selected a single probe for each gene. We made this selection based on the similarity of coexpression patterns for the same gene in the RNAseq coexpression data. For the microarray platforms of *M. truncatula* (Mtr-m) and *P. trichocarpa* (Ppo-m), reliable RNAseq coexpression data were not available. For these two platforms, the expression patterns of multiple probes were averaged to generate a single pattern of expression for that gene. After the selection of probes, a gene-to-gene correlation matrix, which is identical to the probe-to-probe correlation matrix, was calculated by PCC, and this was then converted to the MR index, $MR_{ij}=\sqrt{R_{ij}R_{ji}}$, where $R_{ij}$ indicates that gene *j* is the $R_{ij}$-th strongest coexpressed gene for the guide gene *i* (Obayashi and Kinoshita 2009, Obayashi and Kinoshita 2010).

For RNAseq-based coexpression data, we downloaded the Sequence Read Archive format data from the DDBJ (Ogasawara et al. 2013) and mapped it to NCBI RefSeq sequences (Brown et al. 2015). For this mapping, we employed unique sequence signatures for each gene using Matataki software (https://github.com/informationsea/matataki; Okamura and Kinoshita, in preparation), which enables much faster quantification of RNAseq data than using normal mapping procedures. After conversion to a base-2 logarithm with a pseudo-count of 0.125, ComBat normalization was applied (Johnson et al. 2007), and the average expression levels were subtracted for each gene and experiment. Using all of the experimental data simultaneously, PCCs between all gene pairs were calculated, and these values were then converted to the MR index.

### Bootstrapping procedure for gene coexpression data

To characterize the MR index, we conducted a bagging procedure for gene coexpression data. One bootstrap coexpression dataset was calculated based on randomly selected experiments with replacement to generate the same number of samples as the original dataset. In this study, we repeated this bootstrap calculation of coexpression 100 times for every coexpression platform. The coexpression values between any gene pair should vary with a particular mean and deviation depending on the randomly selected sample. In general, the bootstrap distribution can be used as an estimate of the population distribution and thus can be used to estimate confidence intervals for the value. In addition, the mean of the bootstrapping coexpression values (bagging MR value) was used as another estimate of the MR index.

### Reproducibility between microarray-based and RNAseq-based gene coexpression data

To measure the reproducibility of gene coexpression data from different coexpression platforms in the same species, we used genes that were in common between the coexpression platforms. Then, the top three coexpression relationships from every analyzed gene were used as the edge for this assessment. This edge criterion is also used in the network drawing functions in ATTED-II. The Jaccard coefficient between the two sets of edges was used as the reproducibility score.

### Discriminative power of gene coexpression data for the identification of gene pairs with common functional annotations

To validate the biological significance of coexpressed gene data, we used the enrichment of gene pairs sharing common functional annotations relative to other pairs. For coexpression data in Arabidopsis, GO Biological Process annotations with <50 genes were used. All of the genes within each dataset were divided into two groups, i.e. gene pairs sharing at least one GO annotation and gene pairs without any common annotation. The difference in the distributions of the degrees of coexpression for the two groups was assessed using the partial AUROC_0.01_, which is the area under a part of the receiver operating characteristic curve with a false-positive rate of < 0.01 (McClish 1989). To improve visibility, the partial AUROC_0.01_ multiplied by the square value of the false-positive rate (10,000 in this study) was used as the function score, so that the function scores using randomized gene–function associations were always 0.5. To apply this evaluation method for coexpression data to any species in ATTED-II, KEGG pathway annotations downloaded from KEGG FTP were used (Kanehisa et al. 2016) (June 26, 2017). We selected KEGG pathways associated with <100 genes with a relatively high information content. The genes associated with at least one selected KEGG pathway were then used in this assessment.

## Supplementary Data

Supplementary data are available at PCP online.

## Funding

This work was supported by the Japan Society for the Promotion of Science (JSPS) [KAKENHI grant Nos. 24114005, 15K18464 and 16HP7003 to T.O., and 15K20863 to Y.A.].

## Supplementary Material

Supplementary Data

## Abbreviations

AUROC: Area under the receiver operating characteristic curve
GO: Gene Ontology
KEGG: Kyoto Encyclopedia of Genes and Genomes
MR: mutual rank
PCC: Pearson’s correlation coefficien
RNAseq: RNA sequencing
