# ATTED-II in 2018: A Plant Coexpression Database Based on Investigation of the Statistical Property of the Mutual Rank Index

**DOI:** 10.1093/pcp/pcx209

**Published:** 2018-01-10

**Authors:** Takeshi Obayashi, Yuichi Aoki, Shu Tadaka, Yuki Kagaya, Kengo Kinoshita

The above article was published in *Plant Cell Physiol*. 2017; doi:10.1093/pcp/pcx191

This paper was adapted in print and online to correct an error in formula (1). The correct formula is

$$\text{logit}(p_{i})=\;\text{log}(\frac{p_{i}}{1-p_{i}})\text{(1)}$$

The publisher would like to apologise for this error.

