# Supplementary material for: ATTED-II in 2018: A Plant Coexpression Database Based on Investigation of the Statistical Property of the Mutual Rank Index
Source: Plant Cell Physiol. 2017 Dec 4;59(1):e3. doi: 10.1093/pcp/pcx191 (PMC5914358; doi:10.1093/pcp/pcx191)
Supplement: Supplementary Data [file pcx191_pcp-2017-e-00441-file004.pdf]

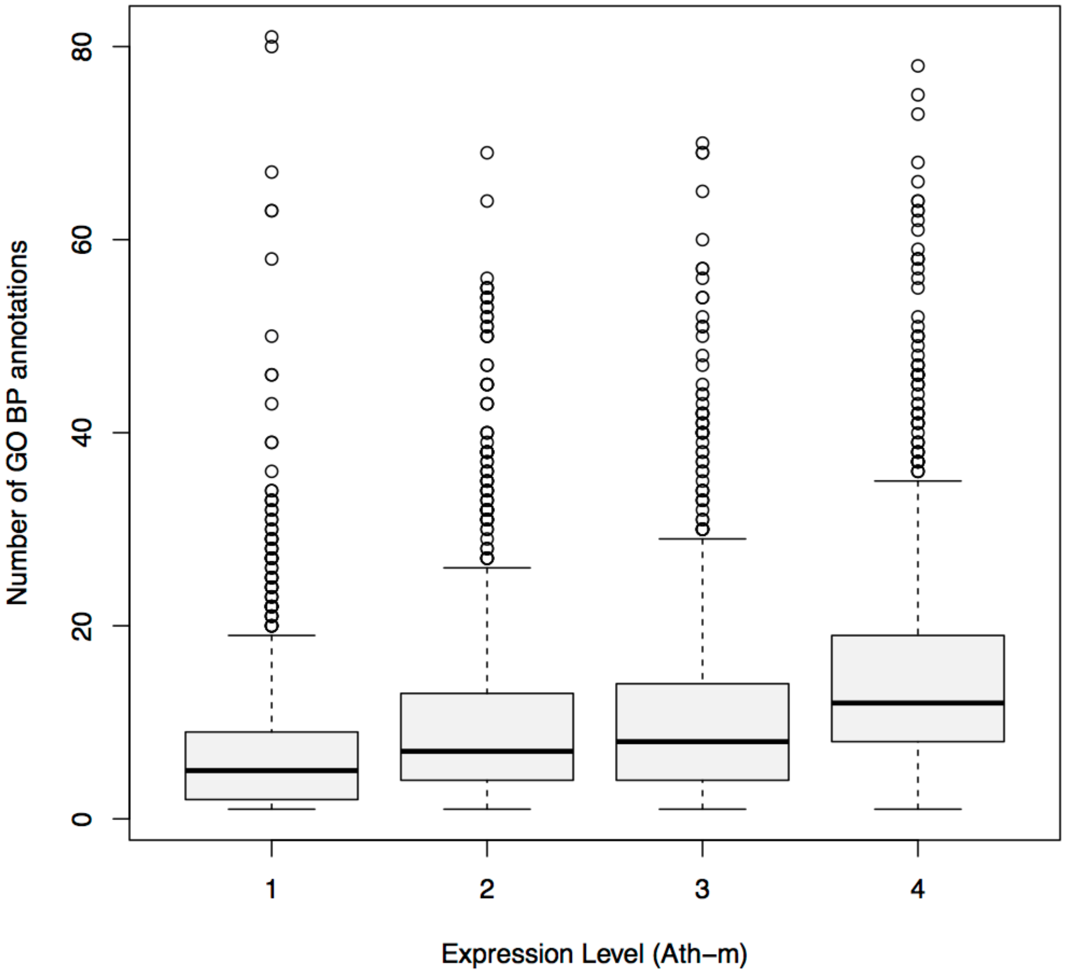

Supplementary Figure 1.
Number of annotations of Gene Ontology Biological Process for each gene depends on the expression level. Arabidopsis genes having at least one Gene Ontology Biological Process terms were divided into 4 levels according to its average expression level on Ath-m. The level 1 is the lowest quarter, while the level 4 is the highest quarter.

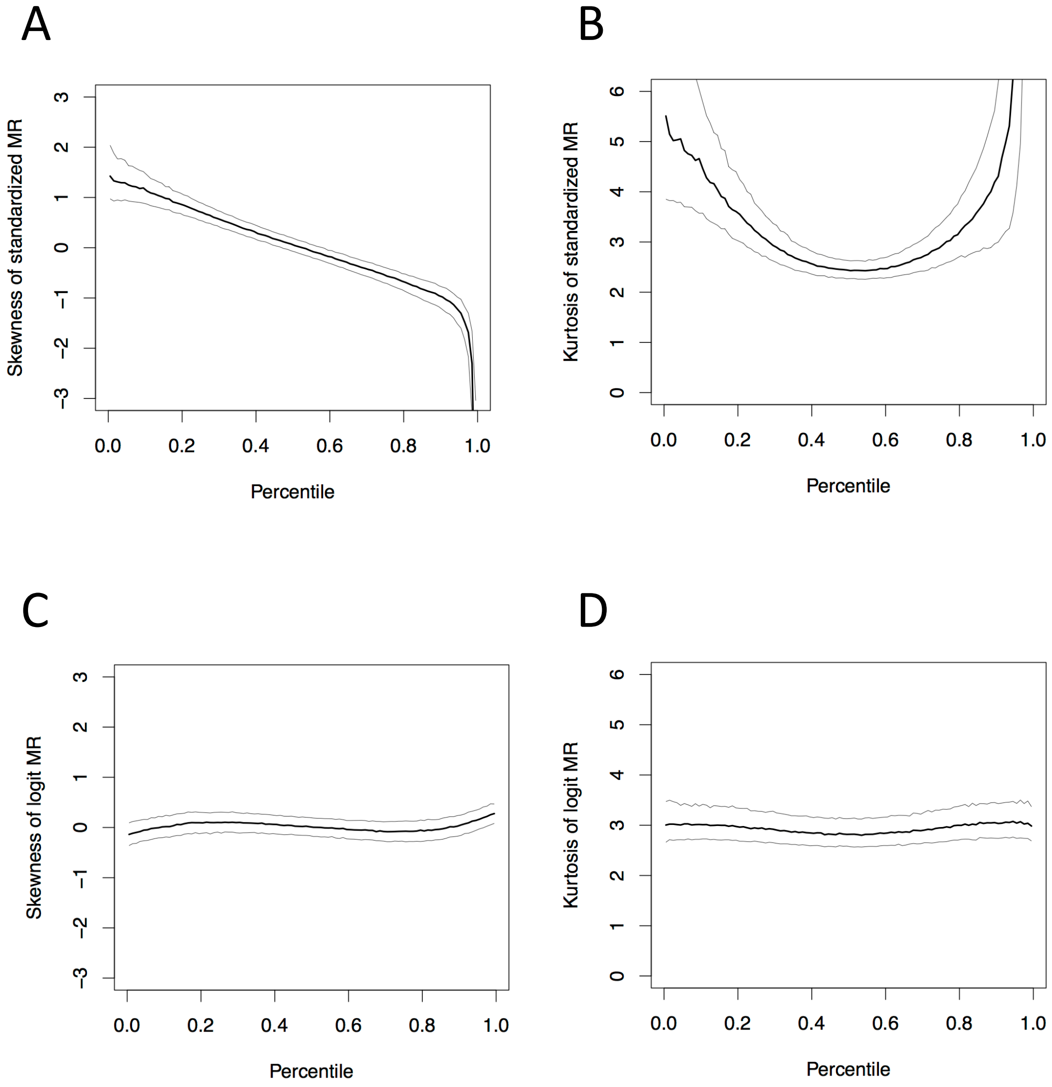

Supplementary Figure 2.

Properties of error distributions of MR and logit-MR values. The error distribution of MR values in RNAseq-based Arabidopsis coexpression data (Ath-r.c3-0) is shown using a bootstrapping approach. The skewness (A,B), and kurtosis (C,D) of bootstrapped MR values (A,C) or logit-MR values (B,D) are shown versus the mean of the bootstrapped MR values. The black lines show the median values with a sliding window corresponding to the 0.01 percentile of the MR without overlap, whereas the gray lines represent the first and third quantiles.
